# Development of a human immuno-oncology therapeutic agent targeting HER2: targeted delivery of granzyme B

**DOI:** 10.1186/s13046-019-1333-6

**Published:** 2019-07-30

**Authors:** Lawrence H. Cheung, Yunli Zhao, Ana Alvarez-Cienfuegos, Khalid A. Mohamedali, Yu J. Cao, Walter N. Hittelman, Michael G. Rosenblum

**Affiliations:** 1 Immunopharmacology and Targeted Therapy Laboratory, Department of Experimental Therapeutics, Unit 1950, The University of Texas MD Anderson Cancer Center, 1515 Holcombe Blvd, Houston, TX 77030 USA; 2 Present address: Department of Pharmaceutical Analysis, School of Pharmacy, Shenyang Pharmaceutical University, Shenyang, 110016 Liaoning China; 3 Present Address: Shenzhen Graduate School, School of Chemical Biology and Biotechnology, Peking University, Nanshan, Shenzhen, 518055 China

**Keywords:** Immunotherapy, HER2, Granzyme B, Kadcyla, Pharmacokinetics

## Abstract

**Background:**

Immunotherapeutic approaches designed to augment T and B cell mediated killing of tumor cells has met with clinical success in recent years suggesting tremendous potential for treatment in a broad spectrum of tumor types. After complex recognition of target cells by T and B cells, delivery of the serine protease granzyme B (GrB) to tumor cells comprises the cytotoxic insult resulting in a well-characterized, multimodal apoptotic cascade.

**Methods:**

We designed a recombinant fusion construct, GrB-Fc-4D5, composed of a humanized anti-HER2 scFv fused to active GrB for recognition of tumor cells and internal delivery of GrB, simulating T and B cell therapy. We assessed the construct’s antigen-binding specificity and GrB enzymatic activity, as well as in vitro cytotoxicity and internalization into target and control cells. We also assessed pharmacokinetic and toxicology parameters in vivo.

**Results:**

GrB-Fc-4D5 was highly cytotoxic to Her2 positive cells such as SKBR3, MCF7 and MDA-MB-231 with IC_50_ values of 56, 99 and 27 nM, respectively, and against a panel of HER2+ cell lines regardless of endogenous expression levels of the PI-9 inhibitor. Contemporaneous studies with Kadcyla demonstrated similar levels of in vitro activity against virtually all cells tested. GrB-Fc-4D5 internalized rapidly into target SKOV3 cells within 1 h of exposure rapidly delivering GrB to the cytoplasmic compartment. In keeping with its relatively high molecular weight (160 kDa), the construct demonstrated a terminal-phase serum half-life in mice of 39.2 h. Toxicity studies conducted on BALB/c mice demonstrated no statistically significant changes in SGPT, SGOT or serum LDH. Histopathologic analysis of tissues from treated mice demonstrated no drug-related changes in any tissues examined.

**Conclusion:**

GrB-Fc-4D5 shows excellent, specific cytotoxicity and demonstrates no significant toxicity in normal, antigen-negative murine models. This construct constitutes a novel approach against HER2-expressing tumors and is an excellent candidate for further development.

## Background

The persistence of immunotherapeutic approaches for control of cancer underscores the longstanding interest in the potential of this modality to achieve a therapeutic advantage using a natural protective immune mechanism. The immune recognition process is a complex interaction [1–7] requiring a number of cofactors and appropriate recognition by various immune cell types [8–11]. The essential mechanism of immune mediated cell killing is initiated by the release of perforin by effector cells which opens transmembrane pores in the target cell. Subsequently, effector cells release GrB which transitions through the transmembrane pores into the target cell cytoplasm. This powerful serine protease causes triphasic, unquenchable cascades resulting in target cell death [12–17]. The exact mechanisms of cytotoxic action of the GrB serine protease have been extensively studied.

We previously described and assessed the in vivo and in vitro efficacy of a number of fusion constructs containing GrB and targeting the Fn14 receptor for TWEAK, the CSPG4 antigen on melanoma, human chorionic gonadotropin (hCG), activated tumor vasculature and HER2 [18–26]. GrB has been well validated as a highly cytotoxic payload with a unique mechanism of action when compared to other payloads employed in targeted therapeutic constructs including antibody drug conjugates (ADCs) and fusion toxins [27–32]. Our observations on the utility of GrB-based constructs have been confirmed by a number of other groups [33–40]. In addition, there are several recent reviews on the characteristics of numerous GrB-based constructs under development [15, 25, 41].

The HER2/neu extracellular domain is a well-validated target for development of a new generation of agents beyond the initial Herceptin therapeutic antibody. The addition of a highly cytotoxic payload emtansine to Herceptin has resulted in an agent Kadcyla (T-DM1) with excellent clinical therapeutic properties [42–45], as well as a number of follow-on ADC products for a wide-range of therapeutic targets, including new designs and novel payloads with unique mechanisms of action [46–50]. The emergence of resistance mechanisms which limit treatment success with these agents is driving innovation to overcome these issues.

This manuscript examines a new framework which incorporates a humanized anti-HER2/neu scFv (designated 4D5) fused through an IgG heavy-chain fragment to active GrB, named GrB-Fc-4D5. Our studies with the parent construct GrB/4D5, composed of GrB and 4D5, supported previous findings that an endosomal release process may be necessary for Her2/neu–targeted agents [18]. Here, we show that incorporation of an Fc domain to link the GrB and scFv moieties not only eliminated the need for endosomal release, but also resulted in a prolonged serum half-life to provide an enhanced biological effect compared to our previous monomeric and relatively low size construct. The current study extends our initial observations of impressive biological activity of GrB-containing anti-HER2/neu fusion constructs and provides a comparison to the FDA-approved ADC Kadcyla.

## Methods

### Cell lines

All cell lines were obtained from the American Type Culture Collection and maintained in RPMI- 1640 (NCI-N87, MDA-MB-453, MDA-MB-468), DMEM/F12 (MDA-MB-231, A549 and SKBR3), McCoy’s 5A (HT-29 and SKOV3), and Minimum Essential Medium with Earle’s salt (SKMel28, MCF-7, Calu3). All media contained 10% FBS. Cells were grown at 37 °C with 5% CO_2_ at constant humidity. Media and supplements were purchased from Life Technologies, Inc.

### Cell line authentication

Cell lines were tested routinely and found to be free of mycoplasma contamination. Cell lines were authenticated by the MD Anderson Characterized Cell Line Core by short tandem repeat (STR) profiling.

### Plasmid construction, protein expression and purification

The GrB-Fc-4D5 and GrB(S195A)-Fc-4D5 constructs were generated by an overlapping polymerase chain reaction method. Briefly, cDNA encoding human GrB was fused via a (GGGGS) linker to the N-terminus of the coding region of hinge CH2 and CH3 of a human IgG1 heavy chain followed by another (GGGGS) linker to the humanized single-chain VH-218 linker-VL variable fragment of the anti-HER2/neu antibody (huscFv4D5). This framework structure is identical to that of the previously described GrB-Fc-IT4 fusion protein [26] with the exception of the sequence of the binding domain. GrB(S195A)-Fc-4D5 is identical to the parent molecule but contains an enzymatically inactive GrB due to the mutation of Serine 195 to Alanine. The completed constructs were cloned into the mammalian expression vector pSecTag2 (Thermo Fisher Scientific). The vector contains a human IgGκ secretion leader sequence in addition to a cMyc epitope tag, a 6xHis tag and an enterokinase (EK) cleavage site. The proteins were expressed by transient transfection of HEK293E cells and purified by immobilized metal affinity chromatography as previously described [26]. To activate the fusion constructs, the leader sequence was cleaved by exposure of the purified protein to enterokinase overnight at room temperature. Enterokinase and the leader sequence were subsequently removed by ion exchange chromatography.

### Determination of antigen binding affinity by ELISA

The antigen binding affinity (*Kd*) and specificity of GrB-Fc-4D5 fusion protein were evaluated by ELISA on HER2/neu extracellular domain (HER2 ECD, G&P Biosciences). GrB(S195A)-Fc-4D5 and a non-specific GrB-Fc-scFv were tested as controls. Briefly, 96-well ELISA plates coated with HER2/neu ECD were incubated with GrB-Fc-4D5 at various concentrations for 1 h at room temperature. After wash steps, anti-GrB antibody C19 (Santa Cruz Biotechnology, Santa Cruz CA) was added and incubated, followed by the addition of horseradish peroxidase-conjugated goat anti-human immunoglobulin antibody. Absorbance at 405 nm was measured after 30 mins on a Thermomax plate reader at room temperature.

### GrB activity assay

The enzymatic activity of the GrB component of GrB-Fc-4D5 and GrB(S195A)-Fc-4D5 was determined in a continuous colorimetric assay using Ac-IEPD-pNA (N-acetyl-Ile-Glu-Pro-Asp-p-nitroanilide, Merck) as a specific substrate. Enzymatic activity of commercial human GrB (Enzyme Systems Products) or GrB-Fc-4D5 was measured by the change in absorbance at 405 nm on a Thermomax plate reader at room temperature. Increases in sample absorbance were converted to enzymatic rates by using an extinction coefficient of 13,100 cm^− 1^ M^− 1^ at 405 nm. The specific activity was calculated per unit of GrB.

### Internalization and localization analysis

Immunofluorescence-based internalization studies were performed on SKOV3 cells as described previously [20]. Briefly, cells were plated into 16-well chamber slides at a density of 1 × 10^4^ cells/well. Then, cells were treated with GrB-Fc-4D5 at 50 nM for 1 h. Fusion protein left binding to the cell surface was removed by a glycine low-pH wash. The cells were then fixed in 3.7% formaldehyde, permeabilized in 0.2% Triton X-100, blocked with 3% bovine serum albumin (BSA) and immunostained for the presence of GrB with rabbit anti-GrB polyclonal antibody (Abcam, Cambridge MA), followed with Alexa Fluor 488-coupled donkey anti-rabbit IgG. Nuclei were visualized with 4′,6-diamidino-2-phenylindole (DAPI). Internalization of GrB-Fc-4D5 into cells was analyzed under a Nikon Eclipse TS-100 fluorescence microscope. The potential localization of GrB-Fc-4D5 in the endosomal/lysosomal compartment was evaluated by treating A549 or MDA-MB-231 cells with 100 nM GrB-Fc- 4D5 labelled with Alexa Fluor 594 (Molecular Probes, Eugene OR) for 2 h at 37 °C, followed by removal of media and incubation with fresh media containing Lysotracker Blue DND-22 (Life Technologies, Carlsbad CA) for 2 h. Cells were washed with PBS and visualized with Nikon A1Rsi multiphoton/confocal microscope using 405 nm and 561 nm lasers for excitation.

### Western blot analysis

Detection of pAKT, pHER2, caspase-9 activation and PARP cleavage was performed by Western blot according to standard procedures [18]. The primary antibodies were as follows: anti-caspase-9, anti-PARP and anti-pAKT were from Santa Cruz Biotechnology (Santa Cruz, CA), anti-pHER2 from Cell Signaling (Danvers, MA), and anti-β-actin from Sigma (St. Louis, MO).

### In vitro cytotoxicity assays

Log-phase cells were seeded (∼3 × 10^3^/well) in 96-well plates and allowed to attach overnight at 37 °C in 5% CO_2_. After 24 h, media was replaced with various concentrations of GrB, GrB-Fc-4D5 or other therapeutic agents at 37 °C for 72 h, in the presence or absence of 5 μM chloroquine. Cell viability was determined using the crystal violet staining method followed by solubilization of the dye in Sorenson’s buffer as described previously [20].

### Pharmacokinetic studies

BALB/c mice at 4 to 6 weeks of age were injected with 200 μg GrB-Fc-4D5. At each time point, three mice were sacrificed. Heparinized blood samples were removed from the chest cavity at 0 (infusion end), 5, 15, 30, and 60 min and at 2, 4, 8, 12, 24, and 48 h after fusion protein administration. The blood samples were then centrifuged and plasma was analyzed for the presence of fusion protein using the GrB capture ELISA method. Samples from each mouse were analyzed in triplicate and plotted as mean ± SEM. Results from determination of fusion protein concentration in plasma were then analyzed by PKSolver to determine pharmacokinetic parameters.

### Toxicology studies in mice

Five female BALB/c mice per group (4–6 weeks old) were injected intravenously every other day (Days 1, 3, 5, 7, 9) for 5 total injections of PBS or GrB-Fc-4D5 (100 mg/kg). One day after the last injection (Acute group, Day 10) or 4 weeks after last injection (Recovery group, Day 38) the mice were euthanized using carbon dioxide and blood was collected via cardiac puncture for hematology and serum chemistry. Histological analyses on tissues obtained at necropsy were acquired and all histological and pathological analyses were performed by the Department of Veterinary Medicine and Surgery at the University of Texas M. D. Anderson Cancer Center.

### Statistical analysis

All statistical analyses were done with software described. Data are presented as mean ± SEM. *P* values were obtained using the 2-tailed *t* test with 95% confidence intervals to evaluate statistical significance. *P* < 0.05 was considered statistically significant.

## Results

### Construction, expression, and purification of GrB-Fc-4D5 fusion protein

**Fig. 1:**
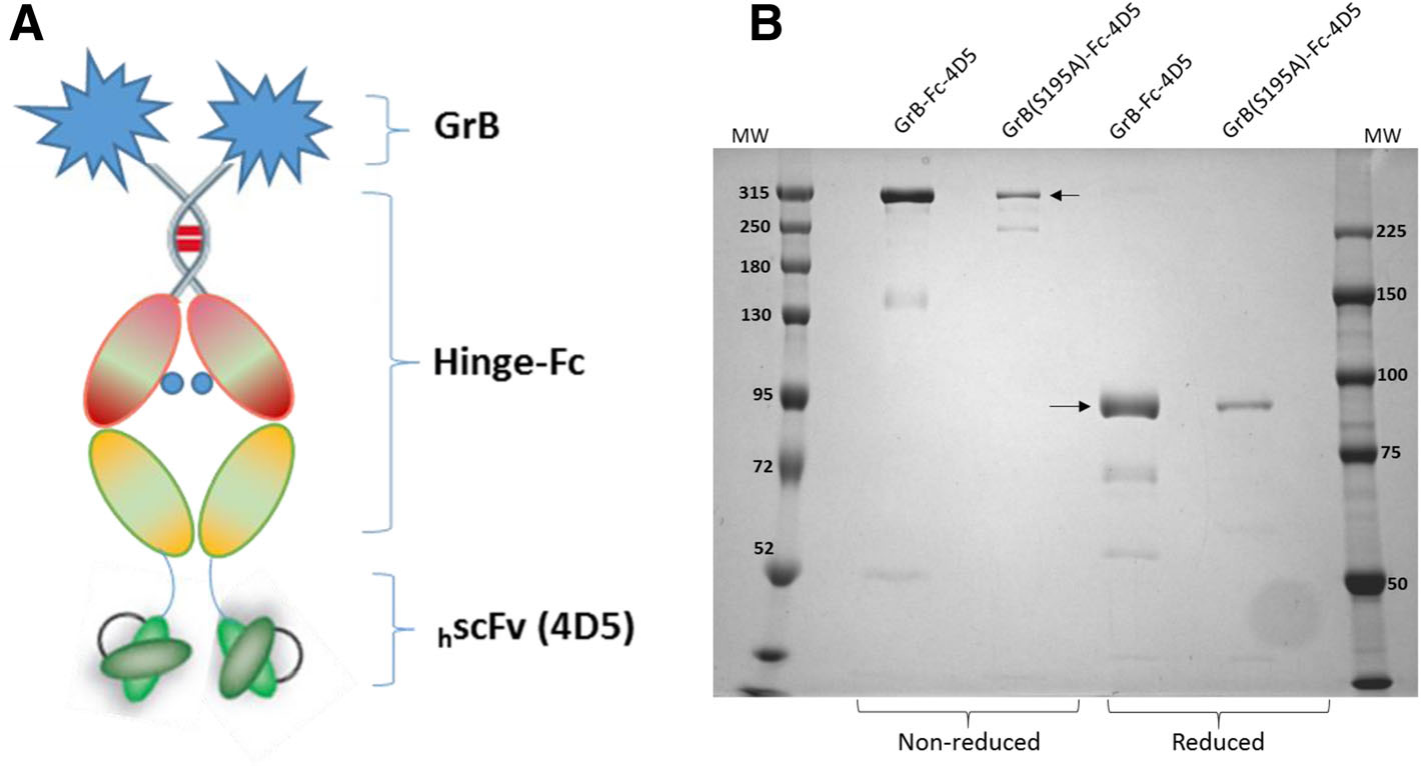
Design and expression of the GrB-Fc-4D5 construct. **a** cDNA of GrB, IgG-Fc region and an anti-HER2 scFv were fused together with a flexible GGGGS linker by overlapping PCR. The resulting plasmid was then cloned into the mammalian expression pSECTag-A vector for either transient HEK-293E or stable CHO expression of the fusion protein. **b** SDS-PAGE of GrB-Fc-4D5 and GrB(S195A)-Fc-4D5 confirmed > 95% purity of the fusion proteins and that both constructs are monomers under reducing conditions and homodimers under non-reducing conditions

Following expression and protein purification through Nickel-IMAC, the fusion protein was incubated with recombinant enterokinase to remove the N-terminal purification tag. The GrB-Fc-4D5 yield following Nickel-IMAC was 100 mg/L, and the final yield was 50 mg/L. The monomer is an 80 kDa polypeptide chain designed for facile dimerization thus creating a dimeric binding domain identical to that of a native IgG and a final molecular weight of 160 kDa (Fig. 1a). SDS-PAGE analysis confirmed the spontaneous dimerization of both GrB-Fc-4D5 and GrB(S195A)-Fc-4D5 under non-reducing conditions (Fig. 1b).

### Analysis of binding affinity

**Fig. 2:**
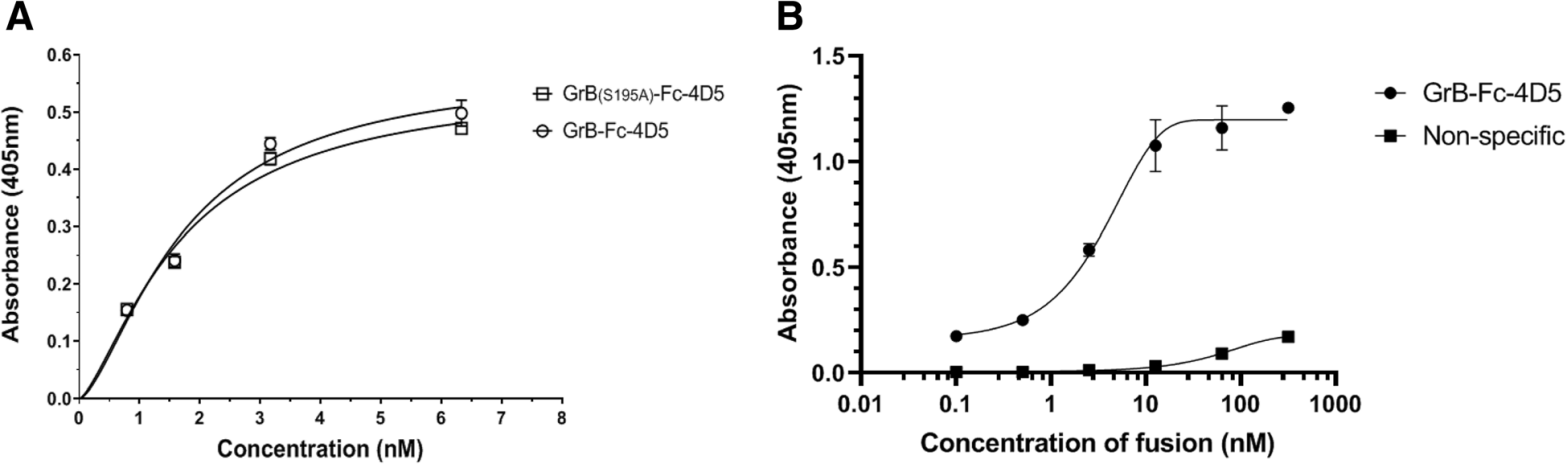
ELISA binding of GrB-Fc-4D5 to Her2/ECD. Purified HER2/neu ECD were coated on 96-well ELISA plates. After incubation with the target protein (1 h) and wash steps, human anti-GrB antibody was added and incubated, followed by the addition of horseradish peroxidase-conjugated goat anti-human immunoglobulin antibody. Absorbance at 405 nm was measured after 30 mins. **a** Various concentrations of either GrB-Fc-4D5 or GrB(S195A)-Fc-4D5 protein to determine K*d* of the 4D5 scFv. **b** Various concentrations of GrB-Fc-4D5 and an unrelated GrB-Fc-scFv (as a specificity control) to confirm specificity of the 4D5 scFv for HER2 ECD

The binding affinities (*Kd* values) of the GrB-Fc-4D5 and GrB(S195A)-Fc-4D5 fusion proteins was assessed by ELISA using purified HER2/neu ECD coated onto a 96-well plate format. The *Kd* values were determined by calculating the concentration of fusion constructs that produced half-maximal specific binding to the HER2/neu ECD. The GrB-Fc-4D5 and GrB(S195A)-Fc-4D5 constructs demonstrated apparent *Kd* values of 1.697 nM and 1.671 nM, respectively, (Fig. 2a) which are in general agreement with the K*d* value of GrB/4D5 [18] and published *Kd* value for native Herceptin (0.150 nM) [51]. Binding of a non-specific GrB-Fc-scFv to HER2/neu ECD revealed negligible binding, indicating specificity of GrB-Fc-4D5 to the HER2/neu antigen (Fig. 2b). As expected, GrB alone did not bind to the HER2/neu ECD (data not shown).

### Enzymatic assay of the GrB-Fc-4D5 fusion construct

To determine the biological activity of GrB-Fc-4D5, we compared the construct’s ability to cleave the substrate Ac-IEPD-pNA to that of native, commercial GrB. The commercial GrB was found to have a specific activity of 371 U/nmol whereas the GrB-Fc-4D5 construct was found to be slightly lower specific activity at 279 U/nmol GrB, indicating that the enzymatic properties of GrB in the fusion protein were retained. As expected, GrB(S195A)-Fc-4D5 was inactive against the GrB substrate.

### Internalization and GrB delivery of the GrB-Fc-4D5 fusion construct

**Fig. 3:**
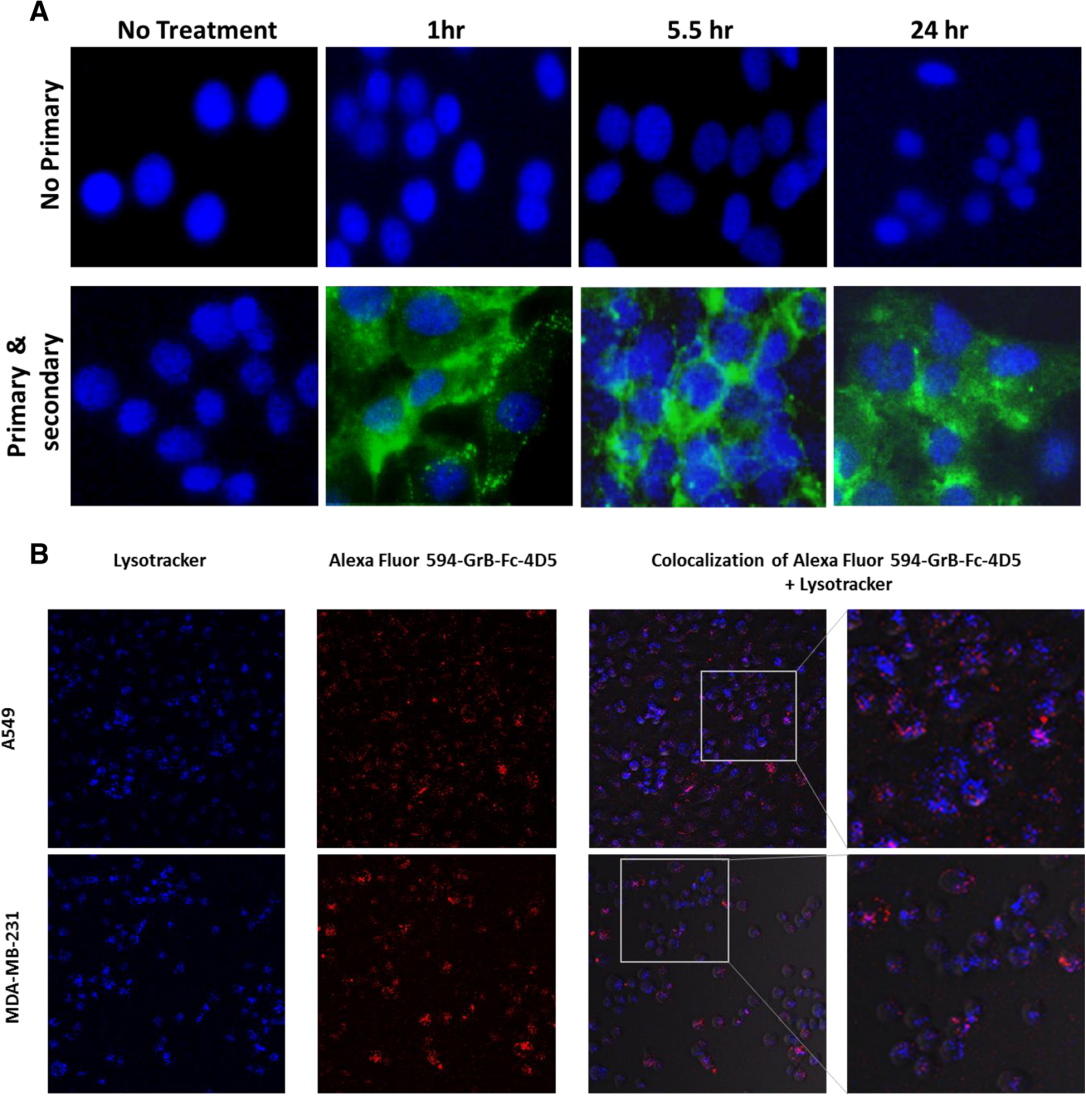
Internalization and localization of GrB-Fc-4D5. **a** Internalization into SKOV3 target cells assessed by a polyclonal anti-GrB antibody demonstrated rapid internalization. SKOV3 cells were either untreated or treated with 50 nmol/L of GrB-Fc-4D5 for 1, 5.5 and 24 h. The cells were fixed, acid washed to remove surface bound fusion protein, and then permeabilized and immunostained for the presence of GrB by using a polyclonal rabbit anti-GrB antibody (green). Cells were counterstained with DAPI (blue) to identify nuclei and visualized using a fluorescent microscope. **b** Intracellular localization of GrB-Fc-4D5. A549 (top) and MDA-MB-231 (bottom) cells were treated with AF-594-GrB-Fc-4D5 (red) for two hours followed by addition of Lysotracker dye (blue). Cells were imaged live by confocal microscopy. Magnification, 20x

Immunofluorescence staining was performed with HER2/neu positive SKOV3 cells to detect internalization and localization of GrB-Fc-4D5. The GrB component of the fusion construct was observed primarily in the cytosol of cells treated with the fusion protein. As seen in Fig. 3, GrB-Fc-4D5 rapidly internalized and efficiently delivered the GrB moiety to the cytosol of the targeted Her2/neu-positive cells, even at the 1 h time point. The degree of internalization appears comparable to that of GrB/4D5 [18]. Both punctate and diffuse staining of the construct in target cells was observed, suggesting the presence of the fusion construct in both the cytoplasm and endosomal/lysosomal compartments. We further investigated the degree of localization of GrB-Fc-4D5 in late endosomes/lysosomes using AF-594-labeled GrB-Fc-4D5 and the Lysotracker Blue DND-22 probe, which accumulates in acidic organelles of live cells. Confocal microscopy of live MDA-MB-231 and A549 cells allowed clear identification of lysosomes as well as internalized GrB-Fc-4D5. However, in both MDA-MB-231 and A549 cells, overlap of the two signals appeared to be negligible, confirming minimal GrB-Fc-4D5 compartmentalization into late endosomes/lysosomes in both cell lines (Fig. 3b).

### Cytotoxic effect of GrB-Fc-4D5 against various tumor cell lines

The cytotoxic effect of GrB-Fc-4D5 was assessed against a panel of tumor cell lines expressing various levels of HER2/neu. In addition, we measured the cytotoxicity of the anti-HER2/neu ADC Kadcyla. As shown in Table 1, the IC_50_ of GrB-Fc-4D5 ranged from 27 nM to 211 nM and the sensitivity of cells did not appear to correlate directly with the expression levels of HER2/neu or the intracellular GrB inhibitor PI-9. Kadcyla was extremely cytotoxic against Calu-3, SKBR3 and MDA-MB-453 cells compared to GrB-Fc-4D5, with the latter more potent against MDA-MB-231 cells. IC_50_ values of the two therapeutics were similar for the rest of the cell lines. The GrB control demonstrated general IC_50_ values above 1 μM in concordance with our previous studies. Interestingly, GrB-Fc-4D5 was more potent against all cell lines that were previously also tested with GrB/4D5 [18]. In order to confirm that the cytotoxic effect is due to the presence of enzymatically active GrB we treated a panel of cell lines with both GrB-Fc-4D5 and GrB(S195A)-Fc-4D5. Treatment with GrB(S195A)-Fc-4D5 resulted in IC_50_s similar to that of untargeted GrB, indicating that the cytotoxicity is due to the active GrB component of GrB-Fc-4D5 and not due to either the Fc or 4D5 component (Table 2).

**Table 1:** HER2/neu and PI-9 status and cytotoxic effect of GrB, GrB-Fc-4D5 and Kadcyla on various tumor cell lines

Cell Line	Cell Type	HER2/neu^a^	PI-9^b^	IC_50_ (nM)		
				GrB	GrB-Fc-4D5	Kadcyla
Calu-3	Lung	109	0.5	> 1000	82	0.02
SKBR3	Breast	100	48	> 1000	56	0.02
NCI-N87	Gastric	85	8	> 1000	105	139
MDA-MB-453	Breast	13	0.4	532	32	0.55
SKOV3	Ovarian	11	100	> 1000	211	38
DU-145	Prostate	3.6	3	> 1000	45	152
MCF-7	Breast	3.4	38	> 1000	99	24
SKMEL28	Melanoma	3.4	73	> 1000	67	63
HT-29	Colon	3.0	0.6	> 1000	36	39
A-549	Lung	2.4	31	> 1000	55	78
MDA-MB-231	Breast	1.5	79	793	27	44
A375-M	Melanoma	1.5	27	> 1000	28	28
AAB-527	Melanoma	1.3	39	> 1000	57	25
MDA-MB-468	Breast	1.1	ND	> 1000	64	31

*Abbreviations*: *PI-9* serine protease inhibitor 9

^a^HER2/neu receptor levels were determined by flow cytometry and normalized to the breast cancer cell line SKBR3

^b^PI-9 protein levels were determined by Western blot analysis and normalized to the ovarian cancer cell line SKOV3

**Table 2:** Cytotoxic effects of GrB-Fc-4D5 and GrB(S195A)-Fc-4D5 on various tumor cell lines

Cell Line	IC_50_ (nM)	
	GrB-Fc-4D5	GrB(S195A)-Fc-4D5
SKBR3	43	1494
MDA-MB-453	22	2294
A-549	49	887
MDA-MB-231	40	685

We next assessed the relative sensitivity of a panel of cell lines grown in log-phase and confluent conditions to GrB-Fc-4D5 and Kadcyla. As shown in Table 3, both GrB-Fc-4D5 and Kadcyla showed excellent cytotoxicity against both confluent and log-phase cultures. Both agents were slightly more cytotoxic against log-phase cultures but quiescence did not induce resistance to any of the cell lines, as all cell lines tested remained sensitive to both constructs.

**Table 3:** Cytotoxic effects of GrB-Fc-4D5 and Kadcyla on log phase and confluent tumor cell lines

Cell Line	GrB-Fc-4D5 IC_50_ (nM)		Kadcyla IC_50_ (nM)	
	Log phase	Confluent	Log phase	Confluent
SKBR3	56	64	< 0.04	< 0.04
HT-29	36	127	41	121
MDA-MB-231	27	58	44	79
A-549	55	68	78	76
DU-145	13	25	125	205

Our previous experience with HER2/neu targeted fusion proteins suggested that accumulation of constructs in endosomes or lysosomes can significantly reduce the sensitivity of cells by preventing delivery of the payload to the cytosolic site of action. We next examined the impact of the lysosomotropic agent chloroquine on the IC_50_ of the GrB-Fc-4D5 construct. Treatment of cells with doses of chloroquine alone as high as 5 μM had no effect on cell viability. As shown in Table 4, the IC_50_ values of the GrB-Fc-4D5 construct against various target cells were not affected by the presence of chloroquine. This suggests that endosomal or lysosomal trapping does not appear to play an important role in the response of cells to the fusion construct, and supports data shown earlier (Fig. 3b).

**Table 4:** Cytotoxic effect of GrB-Fc-4D5 on various tumor cell lines in the presence or absence of the lysosomal inhibitor Chloroquine

Cell Line	IC_50_ (nM)	
	GrB-Fc-4D5	GrB-Fc-4D5 + Chloroquine (5 μM)
SKBR3	44	45
MDA-MB-453	46	49
SKOV3	70	68
DU-145	52	52
A-549	35	40

### Mechanistic studies of GrB-Fc-4D5

**Fig. 4:**
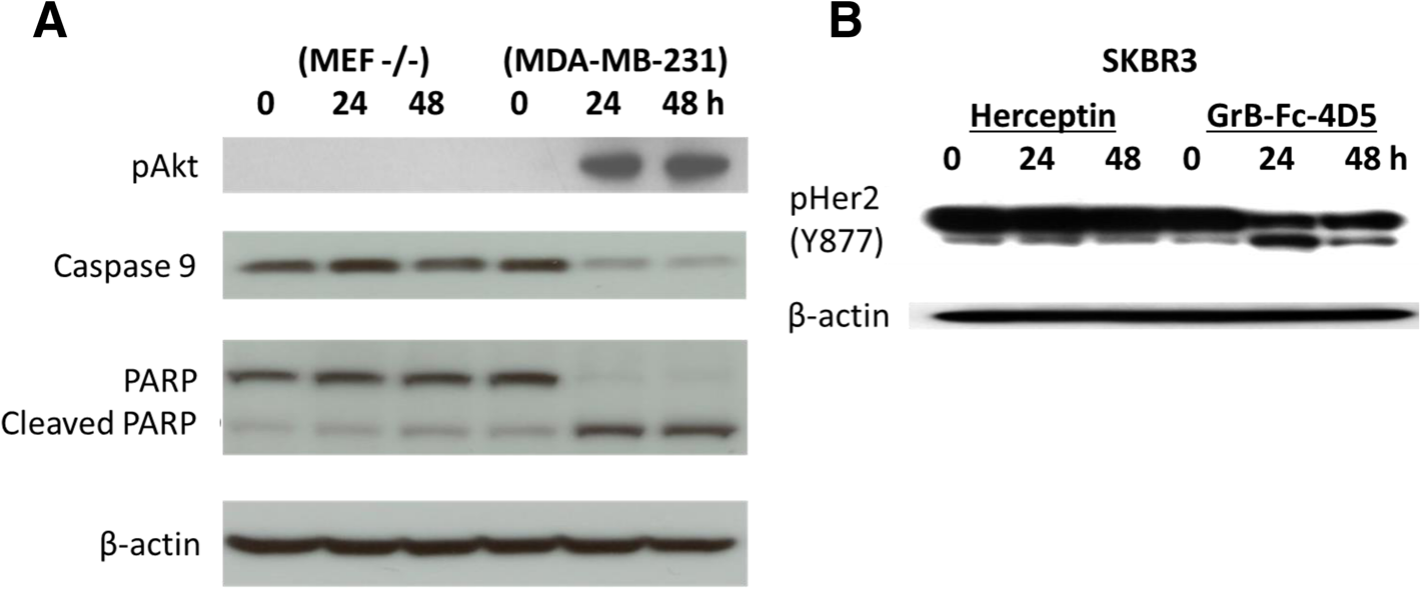
Mechanistic studies of GrB-Fc-4D5. **a** Western blot analysis of MEF3.5^−/−^ (antigen negative) and MDA-MB-231 (targeted) cell extracts for pAkt, Caspase 9 and cleaved PARP after treatment with 20 nmol/L of GrB-Fc-4D5 for 0, 24 and 48 h. **b** Western blot analysis of cell extract for pHer2 (Y877) was assayed against SKBR3 after treatment with 100 nmol/L of either Herceptin or GrB-Fc-4D5 for 0, 24 and 48 h. β-actin was used as a control for protein loading

The mechanism of GrB pro-apoptotic action has been extensively reported by a number of groups, including ours, with respect to targeted fusion constructs. Delivery of GrB to the cytosol generally results in activation of the caspase cascade and release of cytochrome c from the mitochondrial compartment. Both of these events result in rapid activation of cellular apoptosis. We evaluated the mechanism of action of the GrB-Fc-4D5 construct on HER2/neu positive and negative cells. As shown in Fig. 4, treatment with the construct caused an increase in pAkt, cleavage of caspase 9 and cleavage of PARP-1 in MDA-MB-231 (HER2^low^) cells but not in the HER2 negative cell line MEF 3.5^−/−^. Treatment of SKBR3 (HER2^high^) cells with Herceptin showed no effect on phosphorylation of the HER2/neu receptor. In contrast, treatment with GrB-Fc-4D5 resulted in a decrease of phosphorylated HER2/neu after 24 h which persisted for an additional 24 h. These results further indicate that GrB-Fc-4D5 action is not dependent on high levels of HER2/neu expression in order to trigger pro-apoptotic activity.

### Pharmacokinetics of GrB-Fc-4D5 in serum

**Fig. 5:**
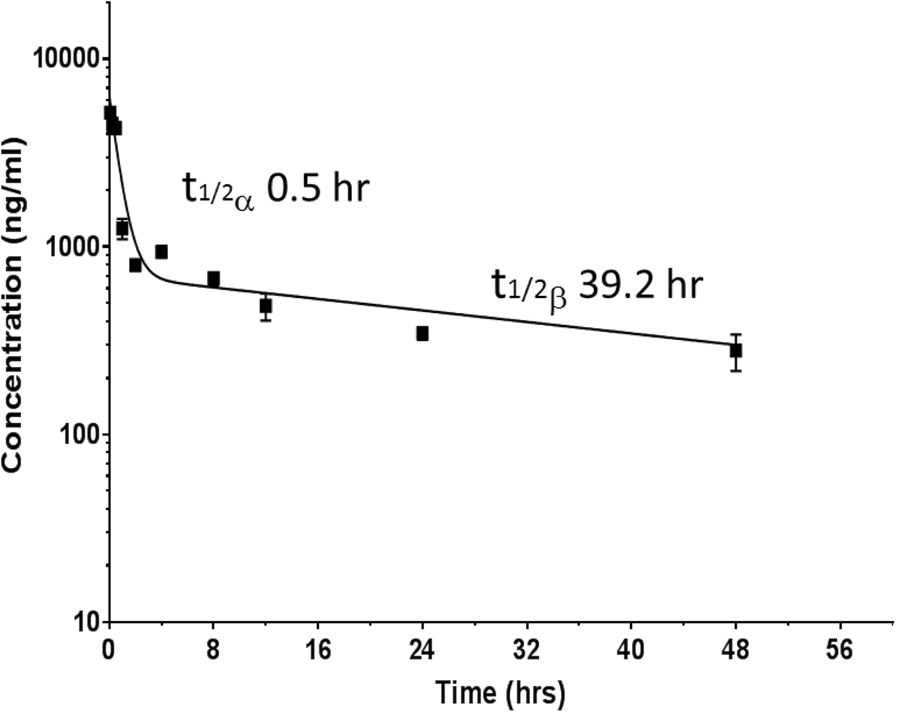
Pharmacokinetic clearance of GrB-Fc-4D5 after IV administration in BALB/c mice. 200 μg of GrB-Fc-4D5 were injected IV into BALB/c mice. Groups of mice (3 mice/group) were sacrificed at various time point after injection. The concentration of fusion protein in plasma was assessed by ELISA according to the protocol in material and method, and the mean blood concentration to time profile of GrB-Fc-4D5 was generated using a least squares nonlinear regression

The pharmacokinetics of the GrB-Fc-4D5 was assessed in BALB/c mice after IV administration. Analysis of the levels of the intact construct in serum was performed using a quantitative ELISA sandwich assay as described. Shown in Fig. 5 are the GrB-Fc-4D5 serum concentrations at various time points after IV injection. The data clearly fit a biphasic clearance curve with an alpha-phase half-life of 0.5 h and a terminal-phase clearance of 39.2 h. The apparent volume of distribution (Vda) of 32 ml suggests distribution of the agent into peripheral sites outside the vascular space.

### Toxicity studies of GrB-Fc-4D5 in BALB/c mice

**Fig. 6:**
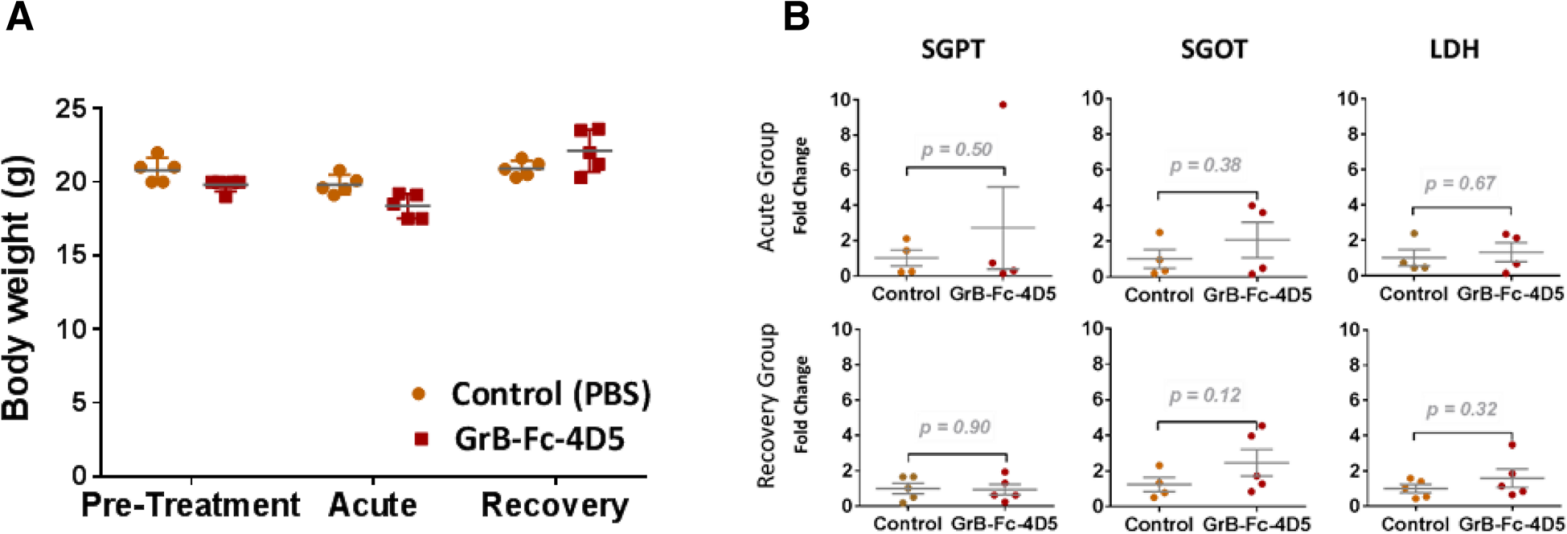
Toxicity assessment of GrB-Fc-4D5 in BALB/c mice. **a** Body weight in control group (PBS) and Grb-Fc-4D5-treated (100 mg/kg) mice. The values represent the weights measured in BALB/c mice before treatment, one day after treatment (acute group) and 4 weeks after treatment (recovery group). **b** Liver enzymes detected in serum of BALB/c treated mice. The enzymatic activity of Alanine Transferase (SGPT), Aspartate transferase (SGOT) and Lactate Dehydrogenase (LDH) was measured in acute and recovery groups. No statistical difference was found between mice treated with control or GrB-Fc-4D5 in the acute or recovery groups

To assess toxicity, BALB/c mice were treated with 5 doses (IV) of the GrB-Fc-4D5 construct administered every other day. Groups of mice (5/group) were sacrificed 1 week after the last injection (acute group) or 3 weeks after the last injection (recovery group). Mice were treated with 20 mg/kg per dose for a total dose of 100 mg/kg. Body weights and serum chemistry studies are shown in Fig. 6a and b respectively, and indicate no statistically significant differences between the treated and control groups. Histopathologic assessment of tissues obtained at necropsy demonstrated a mild generalized inflammatory response in major organs containing lymphoid tissue likely due to administration of a fully human drug product. Otherwise, there were no significant histopathological changes observed between the recovery or acute groups and the control groups (Table 5).

**Table 5:** Macroscopic and microscopic findings in the different organs and tissues extracted from mice in acute and recovery groups

Organ Morphologic Diagnosis	Acute		Recovery	
	PBS	GrB-Fc-4D5	PBS	GrB-Fc-4D5
*Liver/Gall Bladder*	N	A	A	A
Inflammation, chronic, multifocal, interstitium		0.2	0.2	0.2
*Kidneys*	A	N	A	N
Inflammation, chronic, focal, interstitium, pelvis	0.2		0.2	
Karyomegaly & atypia, focal, interstitium				
*Lung*	A	N	N	A
Histiocytosis, focal, alveoli	0.2			0.2
Inflammation, chronic, multifocal, perivascular				0.8
Ectopic tissue, focal, bone, bronchiole				0.2
Embolism, foreign body with inflammation, chronic-active, focal, interstitium				
Adenoma/adenocarcinoma, alveolar-bronchiolar				
*Spleen*	N	A	A	A
Hyperplasia, lymphoid, diffuse, white pulp		1.2	0.4	0.4
*Heart*	N	A	N	A
Inflammation, chronic, focal, interstitium, myocardium		0.6		0
*Skeletal muscle*	N	N	N	N
*Aorta (thoracic)*	N	N	N	N
*Adrenal Glands*	N	A	A	A
Degeneration, X-zone, multifocal, corticomedullary junction		1	1.6	1.8
Hyperplasia, spindle cells, multifocal, subcapsular			1.2	0.8
*Brain*	N	N	N	N
Cyst, epidermal inclusion				
Gliosis, multifocal, neuropil with apoptotic cells				
*Nose*	A	A	A	A
Infiltration, lymphocytes, multifocal, submucosal, nasal mucosa & lacrimal duct	0.8	0.8	0.8	0.2
Degeneration, multifocal, skeletal muscle	0.4			
Regeneration, multifocal, skeletal muscle	0.4			
Malformation, focal, septum & turbinates		0.4		malformation
*Eyes, Ears*	N	N	N	A
Inflammation, acute, multifocal, dermal, bilateral, external ear with mild, acanthosis & hyperkeratosis				0.2
Inflammation, acute, diffuse, bilateral, middle-ear				
*Pituitary Gland*	N	N	N	0
*Sciatic Nerve*	N	N	N	N
*Spinal cord*	A	N	N	A
Degeneration, myofibers, multifocal, paravertebral muscles with mineralization	0.6			
Inflammation, chronic-active, focal, paravertebral skeletal muscle				0.6
*Stomach*	A	A	A	A
Inflammation, acute, multifocal, submucosal	1.2	1.4	1.2	1.8
Hyperkeratosis, diffuse, forestomach with bacterial colonies	1	0.8		
Depletion, Parietal cells, multifocal, mucosa		1.2	1.2	1.6
Metaplasia, intestinal, multifocal, mucosa		0.4		
Hyperplasia, epithelial, multifocal, mucosa				
*Duodenum*	A	A	A	A
Hyperplasia, epithelial, diffuse, mucosa	2	2	1.6	1.4
Inflammation, acute, multifocal, submucosal	0.6	1.8	0.8	1
*Pancreas*	N	N	A	A
Degeneration, acinar cells, focal, parenchyma with chronic inflammation and hemosiderosis			0.4	
Inflammation, chronic, multifocal, interstitium				0.4
*Jejunum*	A	A	A	A
Hyperplasia, epithelial, diffuse, mucosa	1.6	1.8	1	1.6
Inflammation, acute, multifocal, submucosal		1	0.6	1
*Ileum*	A	A	A	A
Hyperplasia, epithelial, diffuse, mucosa	1.2	1.4	1.4	1.4
Inflammation, acute, multifocal, submucosal	0.6	1.2	0.6	1
*Cecum*	A	A	A	A
Hyperplasia, epithelial, multifocal, mucosa	1.2	1.6	1	1
Inflammation, acute, multifocal, submucosal	0.4	1.2	1	0.8
*Colon and Rectum*	A	A	A	A
Hyperplasia, epithelial, multifocal, mucosa	0.8	1	1.2	0.6
Inflammation, acute, multifocal, submucosal		0.6	1	0.4
*Mesenteric Lymph Node*	N	A	A	A
Hyperplasia, lymphoid, multifocal, cortex		0.8	0.2	0.6
*Salivary Gland*	N	N	A	N
Inflammation, chronic, multifocal, interstitium			0.2	
Regeneration, acinar cells, focal, periductular				
*Mandibular Lymph Node*	N	N	N	A
Hyperplasia, lymphoid, diffuse, cortex				0.8
*Thymus*	N	N	N	N
*Thyroid Gland*	A	0	A	N
Ectopic tissue (thymus)	ectopic tissue		ectopic tissue	
*Parathyroid*	A	0		N
Cyst, ultimobranchial	cyst			
Hyperplasia, diffuse, epithelial				
*Trachea*	N	N	N	N
*Esophagus*	N	N	N	A
Hyperkeratosis, diffuse, lumen with bacterial colonies				
Inflammation, focal, submucosal				
*Skin*	A	A		0.8
Dermatitis, chronic-active, multifocal, dermis and epidermis	0.2	0.6		
*Mammary Gland*	N	A	N	N
Inflammation, chronic, focal, periductular				
Hyperplasia, epithelial, focal, subcutaneous		0.2		
*Urinary Bladder*	N	A	N	A
Inflammation, acute, multifocal, submucosal		0.2		
Inflammation, chronic-active, multifocal, submucosal				0.4
*Ovary*	N	N	N	N
*Uterus*	A	A	A	N
Hydrometra, diffuse, lumen	0.6	0.4	0.4	
*Femur/Knee Joint*	N	N	N	A
Inflammation, acute, multifocal, periosteal				0.4
Degeneration, focal, myocytes, skeletal muscle, periosteal				
Regeneration, focal, myocytes, skeletal muscle, periosteal				
Proliferation, focal, synovial membrane				
*Sternum*	N	N	N	A
Inflammation, acute, multifocal, periosteal				0.4

*N* No significant lesion

A = Lesion observed

Grade 1 = minimal, rare < 10%

Grade 2 = mild, infrequent 10–20%

Grade 3 = moderate, frequent 20–50%

Grade 4 = marked, extensive > 50%

## Discussion

The field of targeted therapeutics has been energized by the clinical demonstration of significant response rates and verified clinical benefit in patients with HER2/neu-positive breast cancer treated with Kadcyla in numerous clinical trials [52]. Surprisingly, patients who develop resistance to treatment with the Herceptin antibody alone have been found to be responsive to the drug conjugate and a number of clinical trials are ongoing to combine Kadcyla with other therapeutic agents such as Taxol and anti-PD-1 antibodies [53–58]. However, despite numerous positive clinical trials with Kadcyla, the emergence of resistant phenotypes and the identification of numerous mechanisms permitting emergence of cells resistant to the construct [59, 60] continue to demonstrate that there is room for improvement in the design and development of these targeted therapeutic agents.

Payloads for targeted therapy constructs which do not invoke multidrug resistance (MDR/MRP) mechanisms or which are not dependent on metabolic transformation for biological activation may circumvent the resistance mechanisms identified for Kadcyla [60–63]. The current study employs the highly cytotoxic serine protease GrB as the cytotoxic agent. In contrast to Kadcyla and other ADCs under development, the mechanism of pro-apoptotic action of GrB revolves around activation of the caspase cascade, damage of mitochondria releasing cytochrome c and damage to the DNA matrix. Previous studies in our laboratory have demonstrated that cellular expression of MDR/MRP does not impact the cytotoxic effects of GrB-containing constructs [18]. In addition, these studies demonstrated that resistance to Herceptin has no effect on GrB-induced cytotoxicity. The current study also indicates that another known resistance mechanism for T-DM1, sequestration in the lysosomal compartment, does not appear be a factor influencing GrB-Fc-4D5 cytotoxicity.

We previously showed that the parent construct GrB/4D5 inhibited phosphorylation of HER2/neu at Y877 in BT474 cells [18]. Similar results were observed in this study with GrB-Fc-4D5 with SKBR3 cells. These findings contrast to those observed for Herceptin, which did not block Y877 phosphorylation (this study) or which actually increased phosphorylation of Y877 in both SKBR3 and BT474 cells [64]. GrB-Fc-4D5 also markedly increased caspase-9 activation and inhibited AKT phosphorylation, key events in HER2/neu signaling, compared to GrB/4D5. These findings suggest that GrB-induced apoptosis or other GrB- or Fc-induced events may play a role in the overall cytotoxic effect of this molecule. While addition of the Fc domain has been shown to improve circulation due to dimerization [26], we have previously observed that dimeric anti-HER2/neu constructs deliver cytotoxic payloads more efficiently to the cytosol than monomers [18]. GrB-Fc-4D5 also showed improved delivery to the cytosol compared to the monomeric GrB/4D5. The Fc domain may result in a conformation change that facilitates more efficient endosomal release. Alternatively, the presence of disulfide bonds in the Fc domain may mimic peptides that improve endosomal escape [65].

This study was the first to directly compare the in vitro biological activity and potency of the GrB based construct with that of Kadcyla against a number of tumor cell lines. As shown in Table 1, Kadcyla was much more potent than the GrB-Fc-4D5 construct against three cell lines (Calu-3, SKBR3 and MDA-MB-453) while the GrB construct was slightly more active than Kadcyla against MDA-MB-231. Of note, both MDA-MB-453 and MDA-MB-231 cells have been characterized here and by others as Her2/neu^low^, and resistant to trastuzumab treatment [66–69]. Against the other cell lines, the two constructs showed essentially equivalent potency in the mid-nanomolar range.

There have been a number of GrB-containing constructs generated by other laboratories and these constructs have employed a number of targeting molecules including peptides and single-chain antibodies [33, 35–37, 70–74]. The current construct contains a scFv as the targeting component but the construct is dimeric and has a relatively high molecular weight (~ 160 kDa exclusive of glycosylation). The dimeric, bivalent design was intended to prolong serum half-life and provide an enhanced biological effect in vivo compared to our previous monomeric and relatively low size GrB/4D5 constructs (~ 60 kDa) [18]. Our studies indicate a half-life of 39 h, which can provide a prolonged therapeutic benefit similar to that of native IgG therapeutics. However, the clearance rate is 2–3 times faster than Kadcyla, which may be due to the different species used for pharmacokinetic analysis, and using an alternative analytic method to determine serum concentration [75].

Toxicity studies of the GrB-Fc-4D5 construct were performed after 5 repeated IV injections using an every other day schedule. Although we found no informative toxicity in any of the organs systems studied, it should be noted that the targeting 4D5 single-chain does not bind to the murine homolog of HER2/neu. However, this model would be expected to identify toxicity which can occur by non-specific uptake of the GrB-Fc-4D5 protein. Previous studies indicate that human GrB is 30 times more toxic than murine GrB [76].

The majority of cytotoxic payloads employed in current ADCs are generally agents which target DNA-related events such as tubulin inhibitors and/or minor groove binders. Protein-based immunotoxins under development generally fall into the class of protein synthesis inhibitors. The use of GrB in our constructs constitutes a first-in-class series of molecules since GrB operates through a well-described trimodal attack unleashing pro-apoptotic cascade events. In addition, the GrB mechanism of action is completely distinct from that of other targeted therapeutic agents either in the clinic or under development. Finally, the design of our GrB fusion constructs allows the serine protease activity to be completely active in the intact molecule and therefore requires no hydrolytic or enzymatic cleavage release from the targeting carrier in contrast to other ADCs.

## Conclusions

This report characterizes a new biological framework which fuses enzymatically active GrB through an IgG heavy-chain fragment to a scFv targeting the validated HER2/neu therapeutic target. GrB-Fc-4D5 was cytotoxic against Her2/neu^low^ cells resistant to trastuzumab treatment, and showed no toxicity in vivo at relatively high doses. The GrB mechanism of action is completely distinct from that of other targeted therapeutic agents currently in the clinic. This construct constitutes a novel approach against HER2-expressing tumors and is an excellent candidate for further development.

## Data Availability

The datasets used and/or analysed during the current study are included in this article or are available from the corresponding author on reasonable request.

## Abbreviations

4D5: Humanized anti-HER2/neu scFv
ADC: Antibody drug conjugate
BSA: Bovine serum albumin
DAPI: 4′,6-diamidino-2-phenylindole
EK: Enterokinase
GrB: Granzyme B
hCG: Human chorionic gonadotropin
MDR/MRP: Multidrug resistance
TDM-1: Trastuzumab emtansine, Kadcyla
